# AI in radiology and interventions: a structured narrative review of workflow automation, accuracy, and efficiency gains of today and what’s coming

**DOI:** 10.1007/s11548-025-03547-2

**Published:** 2025-11-17

**Authors:** Michael Friebe

**Affiliations:** Faculty of Computer Science, AGH University of Krakow, Healthtech Innovation Laboratory, Kraków, Poland

**Keywords:** Artificial intelligence, Diagnostic imaging, Workflow automation, Deep learning, Interventional radiology, CT, MRI, Cancer screening, Coronary stenting, Cryoablation

## Abstract

**Purpose:**

Artificial intelligence (AI) is rapidly transforming diagnostic and interventional radiology, supported by accelerating regulatory approvals and clinical adoption. Despite progress, integration varies across modalities and procedures. This study is a structured narrative review of four representative workflows—MRI and CT screening, coronary stenting, and liver cryoablation—to quantify automation readiness, accuracy gains, and efficiency improvements. The novelty lies in comparing diagnostic and interventional domains to highlight distinct maturity levels and future opportunities for AI-driven workflow optimization and clinical value creation.

**Methods:**

A structured analysis was performed identifying 43 workflow steps across the four selected procedures. Each step was evaluated for potential automation, accuracy improvement, and ability to provide new clinical insights, considering current availability and projected 2030 maturity. The assessment drew on peer-reviewed literature, FDA approvals, and industry data (2015–2025). A structured taxonomy distinguished between full automation, human-augmented improvements, and novel AI-enabled guidance functions.

**Results:**

Diagnostic imaging showed higher maturity than interventional workflows. Currently, 70% of MRI and 64% of CT steps have available AI solutions, compared to 55% in coronary stenting and 36% in liver cryoablation. By 2030, nearly all steps are expected to be AI-supported. AI achieved up to 94% segmentation accuracy, 95% nodule detection sensitivity, 30–75% scan time reductions, and 30–50% faster reporting. Interventional applications improved catheter navigation, probe placement, and ablation success but still required significant human oversight.

**Conclusions:**

AI has already demonstrated measurable gains in diagnostic accuracy, efficiency, and workflow standardization. Interventional applications are emerging, with future growth expected in guidance, robotics, and real-time optimization. Despite progress, key limitations include algorithm generalizability, clinical interpretability, organizational readiness, and regulatory uncertainty. AI will augment rather than replace human expertise, with collaborative human-AI workflows being essential. Future integration efforts must address interoperability, workforce adaptation, and ethical considerations to ensure safe, equitable, and clinically impactful deployment.

**Graphical abstract:**

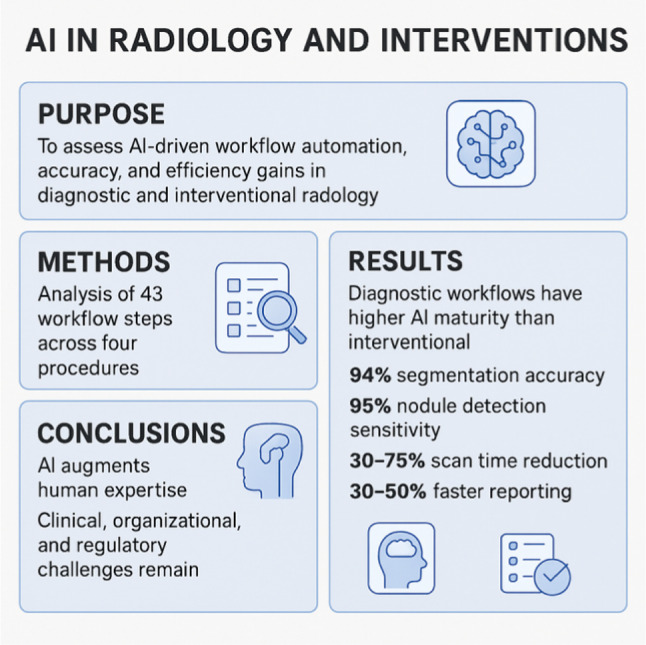

## Introduction

In the last 10 years, there has been a significant increase in AI/machine learning (ML) enabled and regulatory approved (FDA—US Food and Drug Administration) devices and systems that demonstrates robust regulatory acceptance of these technologies. As of August 2025, 1.247 AI-enabled medical devices have received FDA authorization, with radiology devices comprising more than 75% of approvals [1–3]. Health systems increasingly position AI as a practical response to workforce shortages, diagnostic backlogs, care variation, and affordability pressures, driving safe deployment in imaging and image‑guided therapy. Risk‑based frameworks and professional guidance emphasize human oversight, data governance, transparency, interoperability, and lifecycle monitoring, which together explain rapid authorization of AI-based solutions in routine workflows. At the same time, the real‑world evidence base remains heterogeneous and context‑dependent, despite strong technical performance in reader studies and controlled evaluations. Reported error profiles differ between AI and clinicians, reinforcing a collaborative, human‑in‑the‑loop model and the need for external validation, bias assessment, change control, and continuous post‑market monitoring as adoption scales [4, 5].

This regulatory momentum supports continued innovation and clinical implementation [6]. Deep Learning (DL) has become standard in MRI (e.g., GE AIR™ Recon DL and Siemens Deep Resolve) and CT reconstruction (e.g., Canon’s AiCE), associated with improved SNR/low-dose performance and reduced scan time [7–9].

Hardware advances (e.g., photon-counting CT entered clinical use) are often paired with AI post-processing/denoising and generative AI, as reporting assistants (e.g., Nuance PowerScribe Copilot, Rad AI) gain traction for report drafting and administrative automation [10, 11].

The exponential growth in AI applications reflects the technology's maturation from experimental tools to clinically validated solutions that enhance diagnostic accuracy, streamline workflows, and provide new insights into disease detection and management.

AI in that context denotes data‑driven computational systems (mainly machine/deep learning) that reconstruct, analyze, prioritize, and summarize imaging data and guide image‑based procedures under human oversight, meeting risk‑based regulatory expectations (governance, validation, bias/drift monitoring, post‑market control) while delivering automation, improvement, or novel insights whose real‑world impact must be demonstrated prospectively.

The rapid evolution of deep learning algorithms, particularly convolutional neural networks (CNNs), has revolutionized image recognition capabilities, enabling AI systems to detect abnormalities across imaging modalities through automated feature extraction with accuracy matching or exceeding human radiologists [12].

Current applications span from automated image acquisition optimization to complex diagnostic decision support, are fundamentally reshaping how medical imaging procedures are performed and interpreted [13, 14].

This paper responds to that by mapping where AI can responsibly automate, improve, or add new insights across four representative diagnostic imaging and interventional workflows, and by linking opportunities to the safeguards needed for safe, equitable, and economically meaningful scale by 2030.

## Methods

The chosen diagnostic imaging and interventional image-guided procedures are:

1. Cancer screening with MRI (focus prostate and breast),
2. CT lung screening,
3. Coronary stenting procedure, and
4. Ultrasound-guided liver cryoablation.

These procedures across different imaging modalities and clinical specialties came with a total of 43 individual workflow steps (defined by the author after discussion with two domain experts for each of the analyses workflows) that were individually analyzed for their potential for AI automation, improvement capabilities, and capacity to provide new clinical insights. Each step was evaluated for current solution availability and projected 2030 implementation prospects based on technological trends and regulatory developments.

The narrative review followed a structured search and extraction approach aligned to current reporting guidance for medical imaging AI, in which product claims were only used to confirm availability, not performance.

A structured literature scan was conducted across PubMed, Embase, Scopus, IEEE Xplore, and key radiology society resources (RSNA/AJR/JACC/eClinicalMedicine), complemented by regulatory and industry sources for device authorization and deployment context [1–14]. Searches combined clinical terms (MRI, CT, PCI, US, cryoablation, screening, image‑guided therapy) with AI terms (deep learning, machine learning, reconstruction, segmentation, CAD, triage, reporting, navigation) and were limited to 2015–2025 to reflect contemporary practice.

Following definitions were used:

- *Automate*: The step can be executed end to end by an AI system with human oversight, where the residual clinical risk is low and the AI output is directly actionable (e.g., DL reconstruction, automated positioning, standardized report draft generation).
- *Improve*: AI augments a human‑led step by increasing speed, accuracy, or consistency, but a qualified clinician remains the decision‑maker (e.g., CAD triage, segmentation to accelerate PI‑RADS, emphysema/CAC quantification, angio‑derived physiology decision support). Criteria included demonstrated performance benefit with persistent need for clinical judgment..
- *New insights/guidance*: AI provides information not otherwise available or practical at scale (e.g., wire‑free physiology, opportunistic biomarkers on routine CT, intra‑procedural ice‑ball prediction, robotic path planning), typically requiring additional validation of clinical utility and pathway integration before automation is appropriate.

Steps were not labeled ‘automate’ if the action entails high‑stake management changes under uncertainty, requires a patient‑value response, or shows material performance variability across sites/subgroups; such steps defaulted to ‘improve,’ preserving human judgment.

## Results—procedure A—conventional cancer screening using MRI (focus: breast & prostate MRI)

MRI-based cancer screening represents one of the most mature applications of AI in diagnostic imaging, with 70% of workflow steps currently having available AI solutions [15–17]. The procedure involves 10 distinct workflow steps (see Table 1—top procedure), from patient preparation through follow-up planning.

**Table 1:**
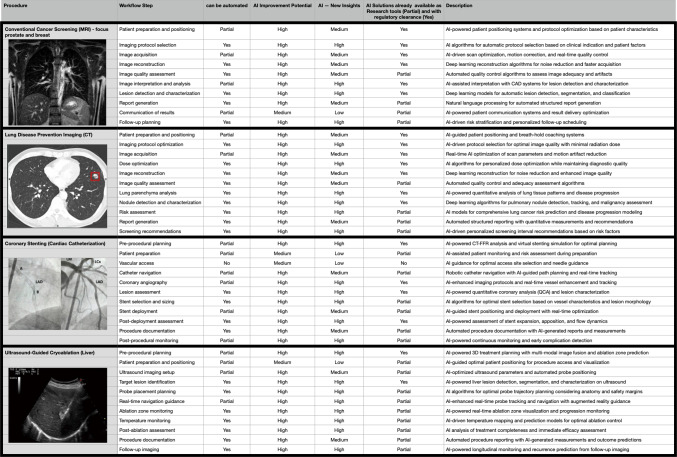
Workflow step analyses (Al automation-/improvement/new insights-potential) of two diagnostic imaging and two interventional procedures. The workflow steps were defined by the author after talking to domain experts. The question on whether it can be automated was based on a personal assessment of the author and supported by technology developments. If Al solutions were already presented as research papers then a PARTIAL rating was given and in the case of commercially available and regulatory cleared products a YES.

Already available high-impact AI applications include automated lesion detection and characterization. The employed deep learning algorithms achieve a 87.6% accuracy in recognizing pathological features, with AI models demonstrating performance comparable to experienced radiologists in breast cancer detection [17]. Also available for that use case are protocol optimization tools, where AI-driven sequence selection reduces scan times while maintaining diagnostic quality, with some systems achieving scan time reductions from 60 to 15 min without compromising image quality.

The subsequent image reconstruction with advanced AI / DL reconstruction techniques significantly reduce noise and improve image clarity, enabling faster acquisition protocols [16].

Several workflow steps can be fully automated, including image reconstruction, quality assessment, lesion detection, and report generation. Four steps show potential for partial automation, particularly in patient preparation and communication of results.

While AI excels at pattern recognition and quantitative analysis, human oversight remains essential for complex clinical decision-making and patient interaction. Automation bias and the potential for missed diagnoses require careful validation and integration strategies [17].

### Evidence & notes

DL MRI reconstruction shortens examinations and improves the signal-to-noise ratio (SNR)—now routine on new systems and many upgrades. Abbreviated breast MRI (AB-MRI) plus AI triage can safely dismiss a sizable share of normal examinations in studies, reducing reads; for prostate MRI, FDA-cleared tools assist PI-RADS workflows and segmentation. Generative AI is increasingly used to draft reports and standardize impressions [18, 19].

## Results—procedure B—lung disease prevention imaging using CT (low-dose computed tomography—LDCT—programs + 'opportunistic' prevention)

CT-based lung screening demonstrates the highest automation potential among analyzed procedures, with almost 3/4 of workflow steps amenable to full automation. This procedure encompasses 11 workflow steps (see Table 1—second procedure), focusing on early disease detection and risk stratification.

Breakthrough AI capabilities include pulmonary nodule detection, which demonstrate superior sensitivity in detecting small pulmonary nodules compared to human radiologists, with some algorithms achieving detection rates exceeding 95% for nodules ≥ 4 mm. Advanced AI models can also predict disease progression and treatment responses by analyzing longitudinal imaging data, enabling personalized screening intervals [20].

And, AI-based dose optimization algorithms, in combination with image protocol adaptations, achieve radiation dose reductions of 36–70% without compromising diagnostic quality, with some pediatric applications achieving up to 95% dose reduction.

AI-powered systems provide precise quantification of disease extent, with automated Interstitial Lung Disease (ILD) quantification showing strong correlation with pulmonary function tests [21]. These tools enable objective monitoring of disease progression and treatment response assessment.

### Evidence & notes

The United States Preventive Services Taskforce (USPSTF) recommends annual LDCT for eligible adults; AI adds value at acquisition (DL recon at ultra-low dose), detection (coronary artery disease—CAD), quantification (volumetry, emphysema), and opportunistic prevention (automated coronary artery calcification (CAC) scoring on routine chest CT and prediction of non-coronary events). Several FDA-cleared CAD products exist; prospective and real-world evidence continues to accumulate [21, 22].

## Procedure C—coronary stenting (PCI) with cardiac cath lab

Cardiac catheterization procedures present unique challenges for AI integration due to their interventional nature, yet show significant potential for workflow enhancement. Currently, 55% of workflow steps have available AI solutions (see Table 1—third procedure), with substantial improvements expected by 2030, also by integrating and using other integrated diagnostic imaging tools, like OCT [23–28].

### Advanced AI applications

Fractional flow reserve analysis with a computed tomography unit (FFR-CT) using AI-enabled computation from CT angiography demonstrates 91% sensitivity and 82% specificity (on a per‑vessel basis versus invasive adenosine FFR in a prospective clinical evaluation using cardiac computed tomography angiography (CCTA) as the anatomical backbone) with studies showing a potential to reduce unnecessary invasive procedures downstream by up to 49% using CCTA pathways incorporating AI‑QCT/FFR‑CT and improved prognostic stratification versus CCTA alone [25].

AI-powered systems also provide real-time vessel tracking and catheter navigation, improving procedural accuracy and reducing radiation exposure [25] and can perform an automated lesion assessment. The DL algorithms for quantitative coronary angiography achieve 87.6% accuracy in pathology recognition, enhancing precision in lesion characterization [24].

Emerging AI-driven robotic systems enable automated catheter manipulation with precision exceeding manual techniques, though full autonomy remains limited. Current systems achieve partial automation in catheter navigation and positioning [26, 27].

Due to its physical nature, placing and inserting a needle-based vascular access, it remains the only step that cannot be automated, requiring manual physician intervention. However, AI guidance for optimal access site selection and needle positioning shows promise for future development [25–27].

### Evidence & notes

AI now guides physiology from angiography (FFRangio/QFR), reaching high diagnostic agreement and similar outcomes vs wire-based FFR in trials/registries; AI-enhanced optical coherence tomography (OCT) (Ultreon) automates calcium detection and vessel sizing; GE AutoRight automates fluoroscopy parameters to balance dose and image quality. Robotic PCI (CorPath GRX) demonstrates reduced radiation and consistent performance in meta-analyses and early clinical experience; full autonomy remains out of scope [28].

## Procedure D—ultrasound image-guided cryoablation of liver tumors

Ultrasound-guided cryoablation represents the most complex procedure for AI integration, with only 36% of workflow steps currently having available solutions but 100% at least partial expected by 2030 [29]. This procedure involves 11 steps requiring real-time imaging guidance and precise temperature control (see Table 1—last procedure).

Emerging AI Capabilities are 3D treatment planning using AI-powered multimodal image fusion, that enables precise ablation zone prediction and optimal probe placement planning. Real-time monitoring with advanced AI algorithms provides continuous ablation zone visualization and temperature mapping, ensuring complete tumor destruction while preserving healthy tissue and ML models analyze treatment parameters to predict ablation success and potential complications, enabling real-time procedure optimization [29].

### Evidence & notes

Computer-assisted stereotactic ablation platforms (e.g., CAS-One IR) improve accuracy and standardization at scale; FDA-cleared robotic systems (XACT ACE; Epione) support guided needle insertion/verification. AI in ultrasound is expanding (segmentation, fusion, needle tracking), with research on contrast enhanced-based recurrence prediction and ice-ball modeling. Cryoablation’s clearer margin visualization complements AI-assisted planning/verification [30–32].

## Accuracy increase and procedure time decrease through the use of AI

AI integration into medical imaging has led to significant improvements both in the accuracy of diagnoses and in procedure times across CT, MRI, and even complex image-guided therapies. Synthesis of predominantly retrospective and an increasing number of prospective real‑world studies shows that AI can improve accuracy and efficiency; however, effect sizes vary and are design‑dependent. The degree of improvement varies by modality and specific workflow, but some broad patterns are evident (see Table 2 for a summary).

**Table 2:** Procedure modality—AI impact on accuracy and procedure timing

Procedure/modality	Accuracy gain With AI	Time reduction With AI
MRI diagnostic screening	90–94% segmentation/artifact correction	30–75% reduced scan time
CT lung nodule screening	+ 8–10% detection (e.g., 93% + sensitivity)	15–40% faster workflow, mins saved
Cardiac/interventional procedures	Improved precision, better targeting	Procedures shorter, fewer re-scans
AI-enabled reporting	Higher consistency, fewer errors	30–50% reduction in reporting time

Meta-analyses and systematic reviews confirm that AI now matches or exceeds experienced radiologists in diagnostic accuracy for detecting disease, often identifying subtle findings that could be missed by humans. For instance, the use of AI systems in PACS (Picture Archiving and Communication Systems) has increased diagnostic accuracy up to 93.2% in some modalities (especially early tumor detection and anomaly identification). Reviews note that convolutional neural networks (CNNs) achieve up to 94% accuracy in image segmentation tasks and are highly effective in artifact correction for CT and MRI [5, 33].

Lung nodule detection (CT): AI detected 8.4% more lung nodules compared to experienced radiologists in patients with complex lung disease, a statistically significant improvement not just in overall accuracy but also in reducing missed subtle lesions [5].

MRI: AI can effectively correct for motion artifacts during MRI, increasing diagnostic reliability especially in populations prone to movement such as children and cardiac patients, and AI-powered accelerated image reconstruction can reduce scan times by 30–50%, and in some protocols up to 75%, translating to faster patient throughput and less motion artifact [34].

Interventional Procedures: In image-guided therapies and interventional radiology, AI provides more accurate probe localization, ablation zone prediction, and real-time guidance, which has improved technical success rates. While hard numbers are less available compared to diagnostic imaging, studies consistently report measurable improvements in correctness of probe placements and ablation completeness [5].

Reviews show that with AI applications two thirds of studies reported a reduction in time required for key clinical imaging tasks such as acquisition, interpretation, and reporting, with some reporting improvements as high as 40% in workflow efficiency [18, 34, 35].

Radiography reporting: A large clinical study found that real-time AI integration led to an average 15.5% increase in reporting efficiency, with some radiologists reaching 40% faster report completion—without loss in accuracy [36, 37].

CT: AI helps optimize scan ranges, automate positioning, and reconstruct images faster, yielding faster diagnostic turnaround —often several minutes saved per patient [18, 38].

Automatic report generation: Natural language processing (NLP) integrated with AI has reduced reporting times by 30–50% and improved consistency, allowing for near real-time generation of structured radiology reports [35, 38, 39].

Critical Condition Triage: For emergent cases like intracranial hemorrhage, AI-enabled triage has reduced time to diagnosis by up to 90% in some hospital systems due to instant alerting and prioritization [33, 36, 37].

The results should however not be overgeneralized as future randomized and multi‑site prospective studies with clinical outcomes are needed to confirm real‑world impact.

## Cross-cutting observations (2015–2025) and 2030 outlook

Acquisition automation is becoming default. DL recon (MRI/CT) and auto-positioning/dose control (CT, angio) are widely deployed and expected to be near-universal by 2030.

From CAD to care orchestration. Beyond detection, AI quantifies disease burden (e.g., emphysema %, CAC) and extracts opportunistic biomarkers to guide prevention, likely embedded in routine chest CT by 2030.

Robotics grows thoughtfully. Evidence supports radiation reduction and consistency. Wider adoption for complex access trajectories (ablation) and standardized PCI steps is also expected, still with human-in-the-loop.

Generative AI for reporting is maturing fastest. Report drafting and follow-up automation are scaling now and by 2030 near-universal availability with strong QA/traceability overlays is expected.

Real-world bias/fairness and generalizability remain active concerns and external validation and standards (CLAIM/FUTURE-AI) are essential for safe scale-up.

For safe scale‑up by 2030, validation standards (external, multi‑site, population‑diverse evaluations with consistent metrics) are essential to demonstrate generalizability and calibrate risk, while continuous post‑market monitoring (bias and drift detection, change control for model updates, real‑time performance dashboards, and periodic re‑validation) sustains reliability across vendors, scanners, protocols, and patient subgroups; together with interoperability and audit trails, these safeguards turn one‑off accuracy into durable, trustworthy clinical impact.

## Discussion

The paper discusses the workflow‑centric synthesis across four distinct pathways, translating heterogeneous literature into an actionable, risk‑aware taxonomy (Automate, Improve, New insights) that links technical maturity to operational governance, validation standards, interoperability, and workforce/consent considerations. By distinguishing product availability from peer‑reviewed performance and collating quantified effects for accuracy, time, and dose, it offers pragmatic guidance for near‑term deployment and a realistic 2030 roadmap. Its limitations stem from evidence heterogeneity (many retrospective or single‑site studies, fewer multi‑site prospective outcome trials), incomplete coverage of all subspecialties, and potential variability across vendors, scanners, protocols, and populations that may attenuate real‑world impact. Outcomes beyond accuracy and efficiency, clinician workload sustainability, patient‑reported outcomes, and cost‑effectiveness, remain less mature and warrant prospective, multi‑site evaluation with bias/drift monitoring and model change control to ensure generalizability and durable benefit.

Analysis across all four procedures reveals significant variation in AI solution maturity. Diagnostic imaging procedures (MRI and CT) show higher current availability (70 and 64% respectively) compared to interventional procedures (55 and 36%). This disparity reflects the greater complexity of integrating AI into real-time procedural environments compared to post-processing image analysis applications [37–39].

Key findings include the potential for full automation in 60% of current workflow steps, with an additional 37% showing potential for partial automation.The only remaining step requires human intervention for safety, ethical, or regulatory reasons. This distribution highlights AI's role as an augmentative technology that enhances rather than replaces human expertise. These oversight needs reflect not a technology shortfall alone but intrinsic socio‑technical requirements, e.g., equity monitoring, informed consent, and harm accountability, that anchor the human‑in‑the‑loop boundary.

It is acknowledged however that the taxonomy was intentionally aligned with risk‑based oversight and human‑in‑the‑loop design to reflect current deployment realities.

Research identifies five distinct AI workflow integration patterns [12]:

- *Secondary Reader Systems*: AI serves as a diagnostic support tool, enhancing radiologist accuracy
- *Primary Screening*: AI performs initial case triage, flagging positive cases for human review
- *Workflow Optimization*: AI automates administrative tasks and improves operational efficiency
- *Real-time Guidance*: AI provides procedural navigation and decision support during interventions
- *Predictive Analytics*: AI models forecast outcomes and optimize treatment planning

There are still a lot of implementation challenges as studies reveal that 24.8% of cases contain clinically significant errors in both AI and human-generated reports, though error types differ between human and AI interpretation. This highlights the importance of collaborative human-AI workflows rather than complete automation [34–37].

It was demonstrated however that efficiency improvements with AI implementation are clearly observable, though with considerable heterogeneity across the studies [35, 36].

The transformation of medical imaging AI is primarily driven by advances in deep learning architectures, particularly convolutional neural networks and transformer models. These technologies enable automated feature extraction from complex medical images, identifying patterns invisible to human observation and providing quantitative assessments previously unattainable.

The next step is multimodal AI Integration combining imaging data with genetic, clinical, and environmental information, creating comprehensive diagnostic models that surpass single-modality approaches. This integration represents a significant advancement toward personalized medicine applications.

All this will require more powerful graphics processing unit (GPU) technology and fast cloud computing interfaces to provide the computational power required for real-time AI applications. The development of edge computing solutions enables AI processing directly on imaging devices, reducing latency and improving workflow integration [38].

Standardization efforts from the industry side are essential to guarantee a seamless flow and use of data generated on different devices and with that initiatives will need to focus on interoperability and standardized AI integration protocols, facilitating seamless implementation across diverse healthcare systems. These efforts address current challenges with vendor-specific solutions and data compatibility issues.

Despite remarkable progress, AI systems face some technical challenges including algorithm opacity, data quality requirements, and generalizability limitations. The "black-box" nature of deep learning models raises concerns about clinical interpretability and decision transparency.

Organizational factors are often underestimated while it shows that healthcare workforce adaptation represents the most critical implementation challenge, requiring extensive training programs and cultural change initiatives. Resistance to change, automation anxiety, and skills gaps impede successful AI integration across organizations [35, 40].

And, AI implementation demands substantial initial investments in technology infrastructure, staff training, and workflow redesign. Many healthcare organizations lack the resources and expertise required for successful deployment.

Data privacy and security concerns create additional implementation barriers, particularly in multi-institutional collaborations. Organizations must navigate complex regulatory requirements while ensuring patient data protection and algorithm accountability [38].

Questions regarding clinical responsibility and legal liability for AI-generated recommendations remain unresolved in many jurisdictions. These concerns affect physician adoption and organizational risk tolerance.

Future automation brings system‑level risks (automation bias, domain shift, drift, equity harms, workflow mismatch, and consent gaps) that must be countered by external multi‑site validation with pre‑specified thresholds, human‑in‑the‑loop checkpoints, explainability and auditability, continuous bias/drift monitoring with change‑control and re‑validation, interoperable telemetry, and organizational readiness for consent, training, and incident learning, so that accuracy gains translate into safe, accountable impact at scale.

Also, further research is required about the evidence gaps of future AI implementation in the presented domain and use cases needs to be studied across the short, medium and long-term.

## Future outlook and 2030 predictions

The human being, the clinician in charge of the procedure, is in most instances the real bottleneck and also the reason why certain applications cannot scale. There are of course many regulatory aspects to consider, as well as ethical questions to be answered if AI automation will actually gain momentum. These are not part of this paper, but they need to be addressed rather sooner than later. While it is not believed that the human element will disappear in the short to midterm timeframe there is hope that the upcoming technological developments will lead to a more democratized access with less dependencies on the human expert.

Beyond diagnostic error profiles, sustainable AI adoption in imaging depends on orchestrating socio‑technical enablers:

- Patient interaction and consent pathways that transparently convey uncertainty and alternatives;
- Workforce readiness through training, change management, and role redesign;
- Governance for accountability (bias/drift surveillance, model update/change control, audit trails);
- Secure, interoperable IT integration across PACS/RIS/EHR with performance telemetry;
- And viable reimbursement and procurement models,

So that validated algorithms translate into reliable, equitable, and economically meaningful improvements at scale.

Foundation models and large language models will enable more generalizable AI systems capable of processing diverse medical data types and providing comprehensive clinical support [39]. The next decade will witness the emergence of increasingly autonomous AI systems capable of managing complex multi-step workflows with minimal human intervention. However, human oversight and clinical judgment will remain essential for complex cases and ethical decision-making.

Predictive analytics and personalized medicine applications will become standard components of medical imaging workflows. AI systems will provide real-time risk stratification, treatment optimization, and outcome prediction integrated seamlessly into clinical decision-making processes.

AI-enabled screening programs will achieve population-scale implementation with personalized risk assessment and adaptive screening intervals. These systems will significantly improve early disease detection while optimizing healthcare resource utilization. The global AI in medical imaging market is projected to grow from USD 1.67 billion in 2025 to USD 26.23 billion by 2034, representing a compound annual growth rate exceeding 35%. This growth will drive continued innovation and clinical adoption across healthcare systems worldwide [41].

- *Short-term prediction (2026–2028)*: AI will focus on workflow optimization, quality improvement, and efficiency enhancement in existing diagnostic processes [39]. Implementation of standardized AI integration protocols and comprehensive training programs [42].
- *Medium-term prediction (2029–2031)*: Development of integrated AI platforms combining multiple imaging modalities with clinical data for comprehensive diagnostic support. Emergence of autonomous diagnostic workflows for routine cases.
- *Longer-term prediction (2032 +)*: Achievement of fully integrated AI–human collaborative systems with seamless workflow integration and real-time clinical decision support across all medical imaging applications..
